# Early initiation of breastfeeding and colostrum feeding among mothers of children aged less than 24 months in Debre Tabor, northwest Ethiopia: a cross-sectional study

**DOI:** 10.1186/s13104-019-4094-6

**Published:** 2019-01-29

**Authors:** Bereket Molla Abie, Yitayal Ayalew Goshu

**Affiliations:** 1 Neonatal Intensive Care Unit, Dangila Primary Hospital, Awi Zone, Dangila, Ethiopia; 2 Departments of Midwifery, College of Health Sciences, Debre Tabor University, Debre Tabor, Ethiopia

**Keywords:** Colostrum feeding, Early initiation, Breast feeding, Debre Tabor, Ethiopia

## Abstract

**Objective:**

To assess early initiation of breastfeeding and colostrum feeding practice among mothers of children aged less than 24 months in Debre Tabor.

**Result:**

Two hundred ninety-seven (297) mothers of children aged less than 24 months participated which made the response rate of 98.1%. Among a total of 297 participants, early initiation of breastfeeding was practiced by 76.8% of mothers. Nearly three-fourths (74.4%) of mothers gave colostrum to their index child. The prevalence colostrums feeding and early initiation of breast feeding is low in Debre Tabor. Improving practice of initiation of breastfeeding and colostrums is recommended by counseling women regarding breastfeeding during ANC visit.

## Introduction

Human breast milk is the optimal feeding for all children. According to World Health Organization (WHO) optimal breastfeeding includes early initiation of breast feeding, exclusive breast feeding for 6 month, frequent feeding, continuous breast feeding for 2 years and increase frequency of feeding during illness [1]. WHO defines early initiation of breastfeeding as it is the initiation of breast milk feeding within 1 h after delivery [2].

Early initiation of breastfeeding has different health benefits like increase ability to defense infections, reduce the risk of diarrhea, and increase the survival rate of children [3, 4]. Neonatal mortality can be prevented by 33% if early initiation of breastfeeding is practiced by mothers [5]. Different studies indicated that late initiation of breastfeeding leads to high neonatal morbidity and mortality. According to a systematic review study, infants who initiated breastfeeding after 1 h were 33% at risk of neonatal mortality [6]. A study from Zimbabwe revealed that delayed breastfeeding increases the risk of developing neonatal sepsis within the first 1 week of life [5]. Neonatal morbidity and mortality of infants who didn’t feed breast milk within 1 h is increased by threefold when compared to infants who fed breast milk with 1 h of birth [7–10].

Colostrum is the first milk that is very important for newborns in protecting infections. Since the first milk is rich in immunoglobin G, colostrum has a great role in disease resistance. Many articles reveal that bacterial, viral, fungal and protozoa infection of the newborn baby can be reduced by feeding colostrum. According to different studies, children who didn’t feed colostrum more likely develop many infections, stunting, underweight, and wasting [11–14].

Neonatal morbidity and mortality in Ethiopia is still a major health problem [15, 16]. Studies done in West Gojam and Gondar indicated that neonatal mortality was as high as 18.6 and 48.3 per 1000 live births respectively [17, 18]. In Ethiopia, the majority of (71%) neonatal deaths occurred during the first week of life [19] and the three major causes of neonatal mortality are prematurity, asphyxia, and neonatal sepsis [20, 21]. But this problem can be reduced by early initiation of breastfeeding and colostrums feeding [5–11].

Despite WHO recommends that every newborn baby to feed breast milk within 1 h, early initiation of breastfeeding is poorly practiced by mothers who give a birth especially, in developing countries [22–26]. As different articles indicated initiation of breast milk within 1 h is poorly practiced in Ethiopia [27–30]. Early initiation of breast feeding is practiced by 39.6%, 83.7%, 47.3% and 62.9% of women in Amibara district [27], Dale woreda [28], Gurage zone [29] and Debre Birhan [30] respectively. Studies done in Miza Tepi, Raya, Kombolcha, and Gondar indicated that colostrums feeding practice in Ethiopia is not adequately practiced [31–34].

Since studies were done in Ethiopia to assess early initiation of breastfeeding is not enough and no studies done in the study area, this study was aimed to assess early initiation of breastfeeding and colostrum feeding practice among mothers of children aged less than 24 months in Debre Tabor town. This study will be important for policymakers, health care providers, and stakeholder in order to improve the practice of early initiation of breastfeeding.

## Main text

### Methods

#### Study setting and design

This community-based cross-sectional study was conducted in Debre Tabor from November 01–30, 2017. Debre Tabor is the capital city of South Gondar zone which is one of the nine zones found in the Amhara regional state. Debre Tabor town is found 666 km away from Addis Ababa, the capital city of Ethiopia in the Northwest direction and it far from Bahirdar by 103 km. There are three health centers, three private clinics, and one general hospital found in the town. In addition to this, there are six health extension workers in all the six kebeles (the smallest unit of the woreda) of the town. Before July 2016, the town had four kebeles but starting from July 2016 the town is divided into six kebeles. According to 2017 central statics agency, the town has an estimated total population of 93, 464. Of these total populations, 49, 246 of them were women whereas the rest 44, 218 of them were men. There are around 31, 831 women who were in the reproductive age group and 2, 786 children who were under 2 years age. The total number of households in the town were 14, 088.

#### Participants

All mothers of children aged less than 24 months who lived in Debre Tabor town during the study period were the study population.

#### Sample size determination and sampling procedure

To calculate the required sample size, single population proportion formula (n = (Zα/2)^2^p (1 − p)//d^2^) was used. 73.1% of population proportion of early initiation of breastfeeding [36], 95% confidence interval, and 5% marginal error. Finally, the final sample size found to be 303. All the six kebeles of the town were incorporated in the study and the calculated sample size was proportionally allocated to all kebeles. Census was conducted and households with mothers of children aged less than 24 months were marked. Two thousand five hundred fifty-eight households were marked since there were mothers of children aged less than 24 months in the houses. The sampled mothers were selected by systematic sampling technique. The sampling interval was calculated by dividing the total number of households with mothers of children aged less than 24 months to sample size.

#### Data collection tools and techniques

The data were collected using a pretested an interviewer-administered questionnaire for a period of 1 month. The questionnaire was developed after reviewing relevant literature. It was prepared originally in English and was translated into local language, Amharic for the purpose of data collection and then it was translated back to English again for consistency and accuracy by language experts. The questionnaire had socio-demographic variables, reproductive characteristics, and questions related to early initiation of breastfeeding and colostrum feeding. For data collection, three diploma degree holder nurses were involved under the supervision of the investigators. Two days of training was given for data collectors and supervisors. The data were daily checked manually for completeness and accuracy.

#### Data analysis

The collected data were coded and entered to Epi data software version 3.1. To summarize the data, the entered data were exported to stastical package for social science (SPSS) software version 20. Tables and graphs were used to present the summarized data using frequency and percentage. Mean and standard deviation were computed for continuous variable.

### Result

#### Socio-demographic characteristics

Two hundred ninety-seven (297) mothers of children aged less than 24 months participated which made the response rate of 98.1%. The respondents’ mean age was 26 years with standard deviation of ± 4 years. Less than half of mothers (40.4%) attended college and above whereas 28 (9.4%) of mothers had no formal education. All (100%) of participants were Amhara in ethnicity and most of (82.2%) of participants were Orthodox Christian in religion (Table 1).

**Table 1:** Socio-demographic characteristics of mothers in Debre Tabort, Northwestern Ethiopia, 2017 (n = 297)

Characteristics	Frequency (N)	Percent (%)
Age		
< 30	36	12.1
20–34	178	59.9
> 34	83	27.9
Religion		
Orthodox	262	88.2
Muslim	30	10.1
Protestant	5	1.7
Occupation		
Housewife	148	49.8
Governmental employee	66	22.2
Merchant	44	14.8
Private employee	27	9.1
Student	12	4
Educational status		
Unable to read and write	28	9.4
Able to read and write	48	16.2
Primary school	21	7.1
Secondary school	80	26.9
College and above	120	40.4
Marital status		
Married	282	94.9
Divorced	8	2.7
Widowed	5	1.7
Single	2	0.7
Husband education		
Unable to read and write	10	3.4
Able to read and write	14	4.7
Primary school	40	13.4
Secondary school	70	23.6
College and above	148	49.8
Husband occupation		
Governmental employee	169	56.9
Merchant	80	26.9
Private employee	29	9.8
Farmer	4	1.3
Family size		
1–2	172	57.9
3–4	100	33.7
> 4	25	8.4
Total	297	100

#### Reproductive health history

Of all a total of 297 mothers, the majority (94.6%) of them had antenatal care (ANC) visit for their index child. Of these mothers who had ANC visits, only 160 mothers (53.9%) had four and more ANC visits. Approximately three-fourth (73%) and more than half (58.9%) of mothers were counseled regarding breast feeding during ANC visit and postnatal period respectively. This study revealed that almost all (96.6%) of mothers delivered their index child at health institution (Table 2).

**Table 2:** Reproductive characteristics of mothers in Debre Tabor, Northwestern Ethiopia, 2017 (n = 297)

Variables	Frequency (N)	Percent (%)
Having ANC visit		
Yes	281	94.6
No	16	3.4
Number of ANC visit (n = 281)		
1	3	1
2–3	118	39.7
≥ 4	160	53.9
Counseled about breast feeding during ANC (n = 281)		
Yes	217	77.2
No	64	22.8
Place of delivery		
Health institution	287	96.6
Home	10	3.4
Sex of index child		
Male	158	53.2
Female	139	46.8
Age of index child		
< 6	100	100
6–11	118	118
12–17	47	47
18–23	32	32

#### Colostrums feeding practice and early initiation of breastfeeding

Among a total of 297 participants, early initiation of breastfeeding was practiced by 76.8% of mothers. Forty-four mothers (14.8%) initiated breastfeeding 1 to 3 h after delivery and one mother initiated breastfeeding after 24 h. Mothers who didn’t feed their breast milk within 1 h were asked the reason for not and 46.4% of participants of them said due to fatigue. Nearly three-fourths (74.4%) of mothers gave colostrum to their index child (Table 3).

**Table 3:** Early initiation of breast feeding and colostrum feeding practice by mothers in Debre Tabor, North western Ethiopia, 2017 (n = 297)

Variables	Frequency (N)	Percentage (%)
Initiation of breast feeding		
Within 1 h	228	76.8
1–3 h	44	14.8
3–6 h	13	4.4
6–24 h	11	3.7
> 24 h	1	0.3
Reasons for not feeding within 1 h		
Fatigue	32	46.4
Caesarian section	10	14.5
Baby was separated	8	11.6
I thought I would not have sufficient milk secretions	8	11.6
Due to medical reasons	6	8.7
No/poor secretions	5	7.2
Give colostrums		
Yes	221	74.4
No	76	25.6
Total	297	Total

### Discussion

The finding of our study reveals that the early initiation of breastfeeding was practiced by 76.8% of mothers (CI; 72% to 81.5%). This result is comparable to a study done in Mota which shows that the prevalence of early initiation of breastfeeding is 78.8% [35]. Similarly, a study done in Dembecha district indicates that the prevalence of early initiation of breastfeeding is 73.1% which is almost comparable to our finding [36].

The finding of this research is slightly lower than a study done in the southern part of Ethiopia which shows that early initiation of breastfeeding is practiced by 83.7% of mothers [28]. The reason for lower prevalence of early initiation of breastfeeding in our finding is may be due to the fact that high frequency of mothers whose age was ≥ 35 years. Studies indicate that elder mothers less likely to practice early initiation of breastfeeding. Since age is one of the determinant factors of early initiation of breastfeeding, the difference may be due to this reason. This difference also may be due to the sample size difference. In addition to this, this difference may be due to the fact that cultural beliefs between Debre Tabor town community and Dale community may be different.

However; our finding is too much higher than studies done in Niger [37] and Zimbabwe [38]. The difference between the current study and Niger study may be due to the fact that socio-demographic difference between two studies. In Niger study, more than half (60%) of the participants had no formal education whereas in our study only 9.8% of mothers had no formal education. So, this socio-demographic difference may give higher prevalence of early initiation of breast feeding in our study [27, 29]. This difference is also may be due to the fact that cultural beliefs difference between the communities. In Zimbabwe, mothers prefer to initiate breast feeding when they are pure (after bathing) which may be a major factor for delay. This finding is also lower than studies done in Axum and Gurage zone which show that 41.6% [39] and 43.7% [29] respectively. The difference between Axum study and our study is may be due to the time difference. Early initiation of breastfeeding practice may be increases time to time. The higher prevalence of early initiation of breastfeeding in our study over the previous Gurage study is may be due to the fact that the lower frequency of mothers who delivered their index child at home. In our study, only 3.4% of mothers delivered their index child at home. Mothers who give a birth in a health institution may be counseled about breastfeeding and health care providers may recommend clients to initiate breastfeeding early [35, 40]. The current finding is also higher than studies done in Debre Birhan (62.6%) [30] and South Gondar zone (48.7%) [40]. This difference may be due to time variation and socio-demographic characteristics difference between the current study and previous studies.

In our current study, we also assessed colostrum feeding practice by mothers and the finding revealed that colostrums feeding is practiced by three-fourths (74.4%) of mothers which is similar to a study done in Mizan Tepi (76.2%) [31]. A finding from Gondar shows that colostrum feeding is practiced by 31% of mothers which is too much lower than the current study [34]. This difference may be due to time difference. In addition to this, higher practice of colostrums feeding in our study is may be due to the fact that the current study was conducted among urban mothers whereas the previous study was conducted among rural mothers [27, 29]. In contrary to this, the current study is lower than studies done in Raya Kobo [32] and Kombolcha [33] which show that colostrum feeding is practiced by 86.5% and 88.6% mothers respectively. This difference may be due to the fact that socio-demographic characteristic between the current and the previous study. The difference also may be due to the fact that cultural beliefs may be are not common for all society. Cultural beliefs are more respected in Debre Tabor society than in Kombolcha and Raya because Debre Tabor communities are more bigoted for religion. Assessing the status of early initiation of breast feeding and colostrums feeding in Debre Tabor is very helpful for policymakers and stakeholders in planning maternal and child health care services.

In conclusion, our study concluded that the practice of early initiation of breastfeeding was low when it is compared to WHO recommendation. WHO recommends that every newborn baby has to feed breast milk within 1 h after birth and feed colostrum. This study also confirmed that the practice of colostrums feeding is low in the study area. Improving the practice of initiation of breastfeeding and colostrum is recommended by counseling women regarding breast feeding during ANC visit.

## Limitation

Since this study included mothers whose index child age was up 23 months, recall bias might occurred. Another limitation of this study is participants’ feelings and determinant factors were not studied.

## Abbreviations

ANC: Ante Natal Care
SD: standard deviation
WHO: World Health Organization
